# Transitional changes in gastrointestinal transit and rectal sensitivity from active to recovery of inflammation in a rodent model of colitis

**DOI:** 10.1038/s41598-021-87814-7

**Published:** 2021-04-15

**Authors:** Yan Chen, Yu Guo, Payam Gharibani, Jie Chen, Florin M. Selaru, Jiande D. Z. Chen

**Affiliations:** 1 Division of Gastroenterology and Hepatology, Johns Hopkins University School of Medicine, Baltimore, MD USA; 2 Division of Gastroenterology and Hepatology, University of Michigan, Ann Arbor, MI USA; 3 Division of Gastroenterology, Binzhou Medical University Hospital, Binzhou, Shandong China

**Keywords:** Gastrointestinal diseases, Gastroenterology

## Abstract

Patients with ulcerative colitis are typically suspected of an inflammatory flare based on suggestive symptoms of inflammation. The aim of this study was to evaluate the impact of inflammation on colonic motility and rectal sensitivity from active to recovery of inflammation. Male rats were given drinking water with 5% dextran sulfate sodium for 7 days. Inflammation, intestinal motor and sensory functions were investigated weekly for 6 weeks. (1) The disease activity index score, fecal calprotectin and tumor necrosis factor alpha were increased from Day 0 to Day 7 (active inflammation) and then decreased gradually until recovery. (2) Distal colon transit was accelerated on Day 7, and then remained unchanged. Whole gut transit was delayed on Day 7 but accelerated from Day 14 to Day 42. (3) Rectal compliance was unaffected from Day 0 to Day 7, but decreased afterwards. (4) Rectal hypersensitivity was noted on Day 7 and persistent. (5) Plasma acetylcholine was decreased on Day 7 but increased from Day 14 to Day 42. Nerve growth factor was increased from Day 7 to Day 42. DSS-induced inflammation leads to visceral hypersensitivity that is sustained until the resolution of inflammation, probably mediated by NGF. Rectal compliance is reduced one week after the DSS-induced inflammation and the reduction is sustained until the resolution of inflammation. Gastrointestinal transit is also altered during and after active colonic inflammation.

## Introduction

Ulcerative colitis (UC) is a chronic inflammatory condition characterized by alternating periods of flares and remissions. The most frequent symptoms in active UC are increased frequency of defecation (83%), urgency (85%), a feeling of incomplete evacuation (78%) and tenesmus (63%)^1^. Although the incidence and prevalence of UC have stabilized in high incidence areas such as Western Europe and North America, it is dramatically increasing in the developing countries, such as China and India^2,3^.

Numerous studies have provided evidence that inflammation affects colonic motility and sensory function in active UC, contributing to symptoms commonly seen in UC patients^4,5^. Though treatments for inflammation in UC have become more effective, a significant proportion of patients in remission still suffer from persistent symptoms^6^. Based on these observations, we hypothesized that the persistence of symptoms might be attributed to persistent abnormalities in colorectal motility, compliance and sensation in spite of resolution of inflammation.

Colon motility has been studied in UC patients. Rao et al. found that patients with active colitis had rapid transit through the rectosigmoid region, but patients in remission displayed normal transit^7^. Reddy et al. reported that colonic motility and transit in moderate or mild left-sided UC patients were characterized by increased low-amplitude propagating contractions and variable transit according to a combined manometric/scintigraphic approach^4^. Similarly, Bassotti et al. performed a 24 h colonic manometric study and reported that the propagated contractions were significantly increased in active, moderate UC patients^8^. These data argue that colonic motility is increased and colonic transit is accelerated in active UC. However, there is a need to better understand how colorectal motility and sensation as well as rectal compliance change as a function of resolving inflammation.

Present opinions of visceral sensation in UC patients during active and quiescent phase are not consistent. Rao et al. reported that patients with quiescent UC developed a decrease in rectal sensitivity and an increase in rectal compliance during remission of colitis^9^. Chang et al. found that rectal perception was attenuated in UC patients with mild chronic inflammation of the rectum but enhanced in irritable bowel syndrome (IBS) patients without mucosal inflammation, considering that activation of antinociceptive mechanisms may prevent the development of visceral hyperalgesia in chronic mild inflammation^10^. However, Salameh et al. reported recently that chronic colitis induced visceral hypersensitivity and increased anxiety in the remission period with a low-grade intestinal inflammation in rats^11^. Eduard et al. claimed that symptoms in patients with UC in remission were associated with visceral hypersensitivity and mast cell activity, indicating that increased rectal hypersensitivity is an important factor for IBS-like symptoms in UC patients in remission^12^. Therefore, visceral sensitivity should be investigated in a consecutive way from the active phase to the remission to reveal the effect of inflammation on sensitivity.

Fecal calprotectin has been shown to be a fairly reliable predictor of clinical relapse in UC, which makes the test a promising non-invasive tool for monitoring and optimizing therapy^13^. Plasma tumor necrosis factor alpha (TNF-α) combined with fecal calprotectin and disease activity index (DAI), are adopted to accurately evaluate the inflammation. Distal colon transit time (dCTT) and whole gut transit time (WGTT) are commonly used for the assessment of GI motility. Rectal sensitivity is typically assessed by abdominal electromyogram (EMG) responses to rectal distention. Molecularly, acetylcholine (ACh) is known to play an important role in the regulation of colon motility among other things^14^; Nerve growth factor (NGF) has recently been reported as a major mediator of visceral hypersensitivity^15^.

The present study was aimed to investigate the transitional variations of inflammation on colon motility and sensitivity as well as rectal compliance in the evolution of inflammation from active to resolution in a rodent model of dextran sulphate sodium (DSS)-induced colitis and to explore the correlation of dysmotility and hypersensitivity involving ACh and NGF.

## Results

### Effects of DSS on inflammation

**Figure 1:**
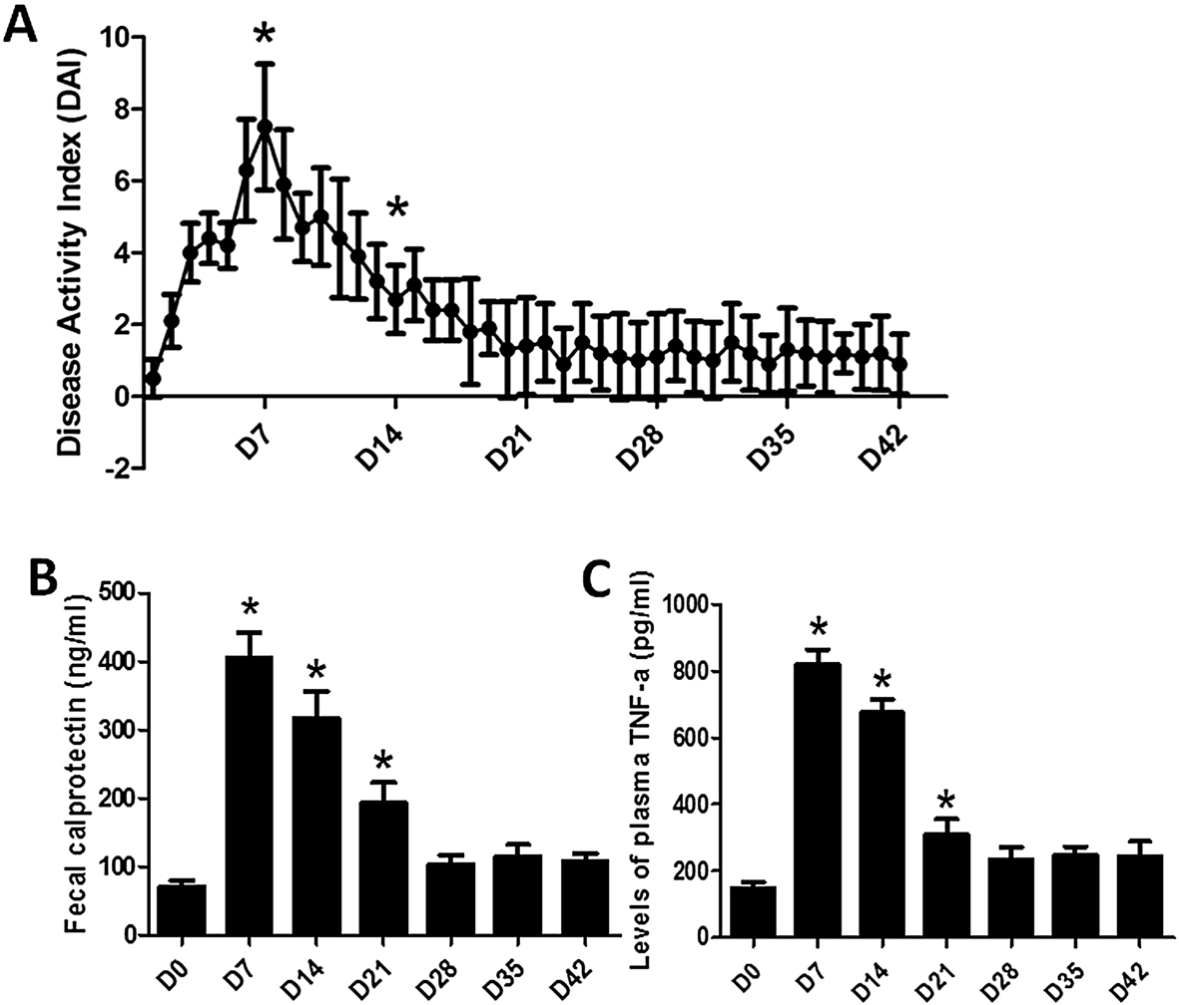
Disease activity index (DAI) (**A**), fecal calprotectin (**B**) and levels of plasma TNF-α (**C**). *P < 0.05 compared with Day 0. (**A**: N = 8, **B** and **C**: N = 6).

(1) *DAI score* The alteration of DAI score was recorded daily in DSS-treated rats during the entire study. As shown in Fig. 1A, the DAI score was significantly increased from Day 1 to Day 7 with administration of DSS water. The DAI score on Day 7 and Day 14 was significantly increased, compared with Day 0 (*P* < 0.001). In the following days without DSS water, the DAI score was gradually decreased and then maintained low, suggesting a spontaneous recovery of inflammation.

(2) *Fecal calprotectin*: Fecal calprotectin was measured for the assessment of inflammation (Fig. 1B). In comparison with Day 0, the fecal calprotectin level was increased significantly on Day 7 (*P* < 0.001), Day 14 (*P* < 0.001) and Day 21 (*P* = 0.002). The fecal calprotectin level was comparable to that before the DSS administration from Day 28 to Day 42, suggesting a complete recovery of inflammation starting from Day 28.

(3) *Levels of plasma TNF-α:* Similar to the findings on fecal calprotectin, the plasma level of TNF-α was significantly elevated from Day 7 to Day 21 (all, *P* < 0.008, vs. Day 0) but returned to the baseline level starting from Day 28 to Day 42 which was consistent with the findings on fecal calprotectin (all, *P* > 0.05 vs. Day 0) (Fig. 1C).

(4) *Histological score:* On Day 42, no difference was noted in the histological score between the normal rats and the rats administrated with DSS (*P* = 0.346).

These results (DAI, fecal calprotectin and TNF-α) revealed that a 7-day administration of DSS induced inflammation that lasted 2 more weeks after the termination of the administration.

### Altered gastrointestinal motility during and after inflammation

**Figure 2:**
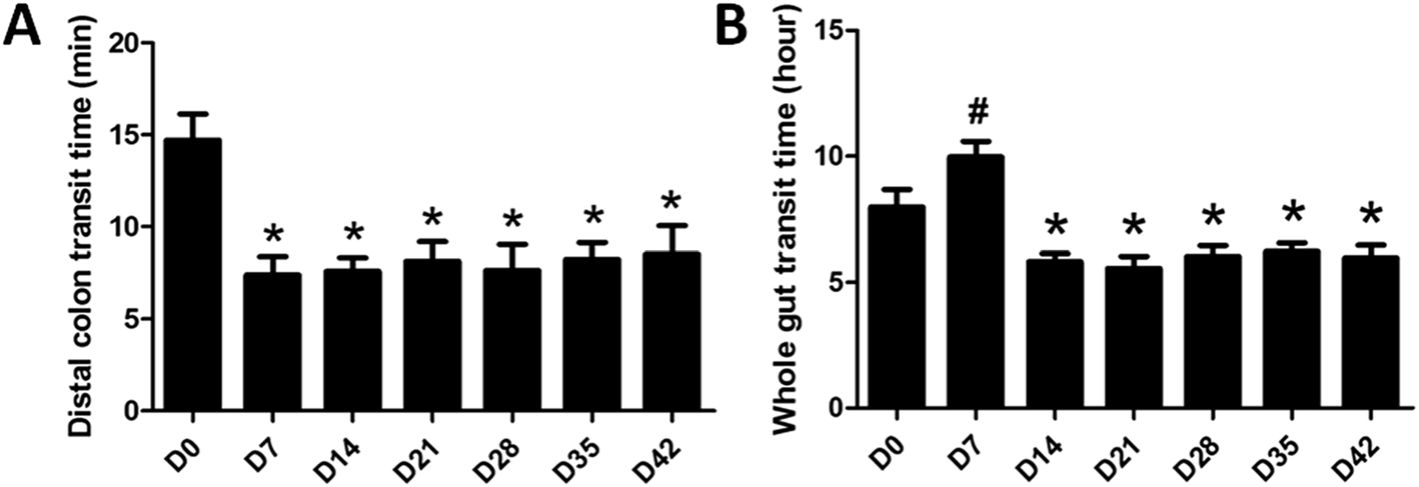
Variation of distal colon transit time (dCTT) (**A**) and whole gut transit time (WGTT) (**B**) in DSS-induced colitis rats. Both * and ^**#**^*P* < 0.05 compared with Day 0. (N = 8).

(1) *Distal Colonic Transit Time*: The dCTT was reduced by half on Day 7 compared with Day 0 (*P* = 0.001) (Fig. 2A), demonstrating an acceleration in distal colon transit. It remained shorter during the subsequent 5 weeks (*P* < 0.02, for all 5 weeks).

(2) *Whole Gut Transit Time*: Interestingly, the WGTT was increased on Day 7 (*P* < 0.005, vs. Day 0), but decreased during the sequent weeks from Day 14 to Day 42 (*P* < 0.02, vs. Day 0) (Fig. 2B).

### Reduced rectal compliance after inflammation

**Figure 3:**
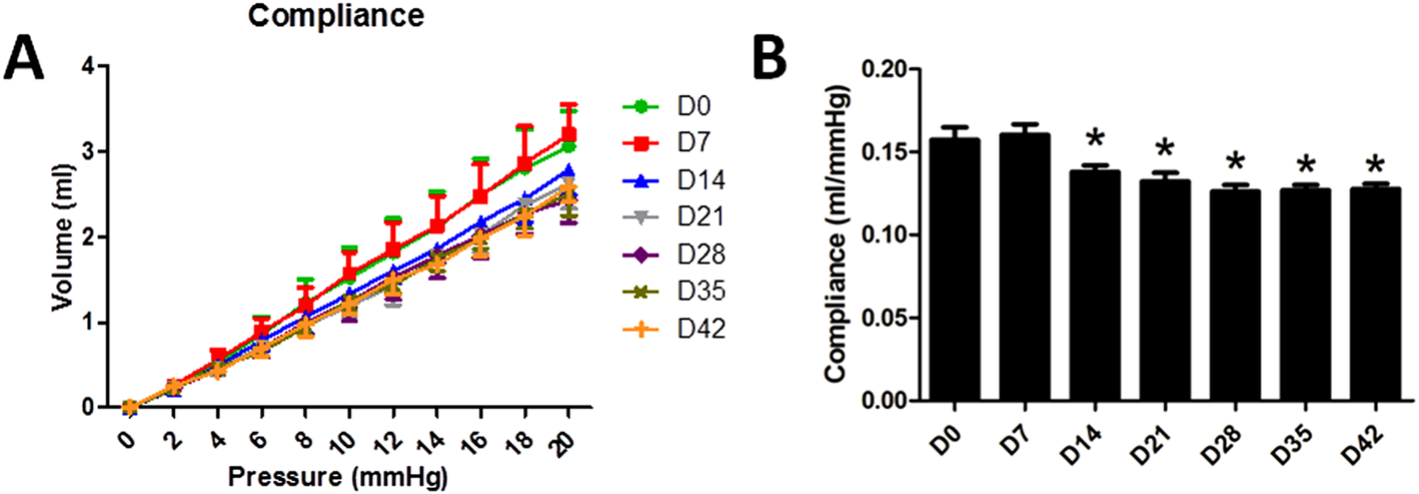
Alterations of rectal compliance in DSS-induced colitis rats. (**A**) The pressure–volume curves from the sequential isobaric distensions at different time points. (**B**) Rectal compliance at different time points. *P < 0.05 compared with Day 0. (N = 8).

The rectal compliance was reduced from Day 14 to Day 42. Figure 3A shows the pressure–volume curves from the sequential isobaric distensions. The rectal compliance, namely the slope of the curves, was not altered immediately after the DSS administration (*P* = 0.667, Day 7 vs. Day 0) but significantly reduced on Day 14 (*P* = 0.009, Day 14 vs. Day 0); the reduction remained during the subsequent weeks (*P* < 0.001) (Fig. 3B). These results showed a reduced rectal compliance after the 7-day administration of DSS.

### Visceral hypersensitivity during and after inflammation

**Figure 4:**
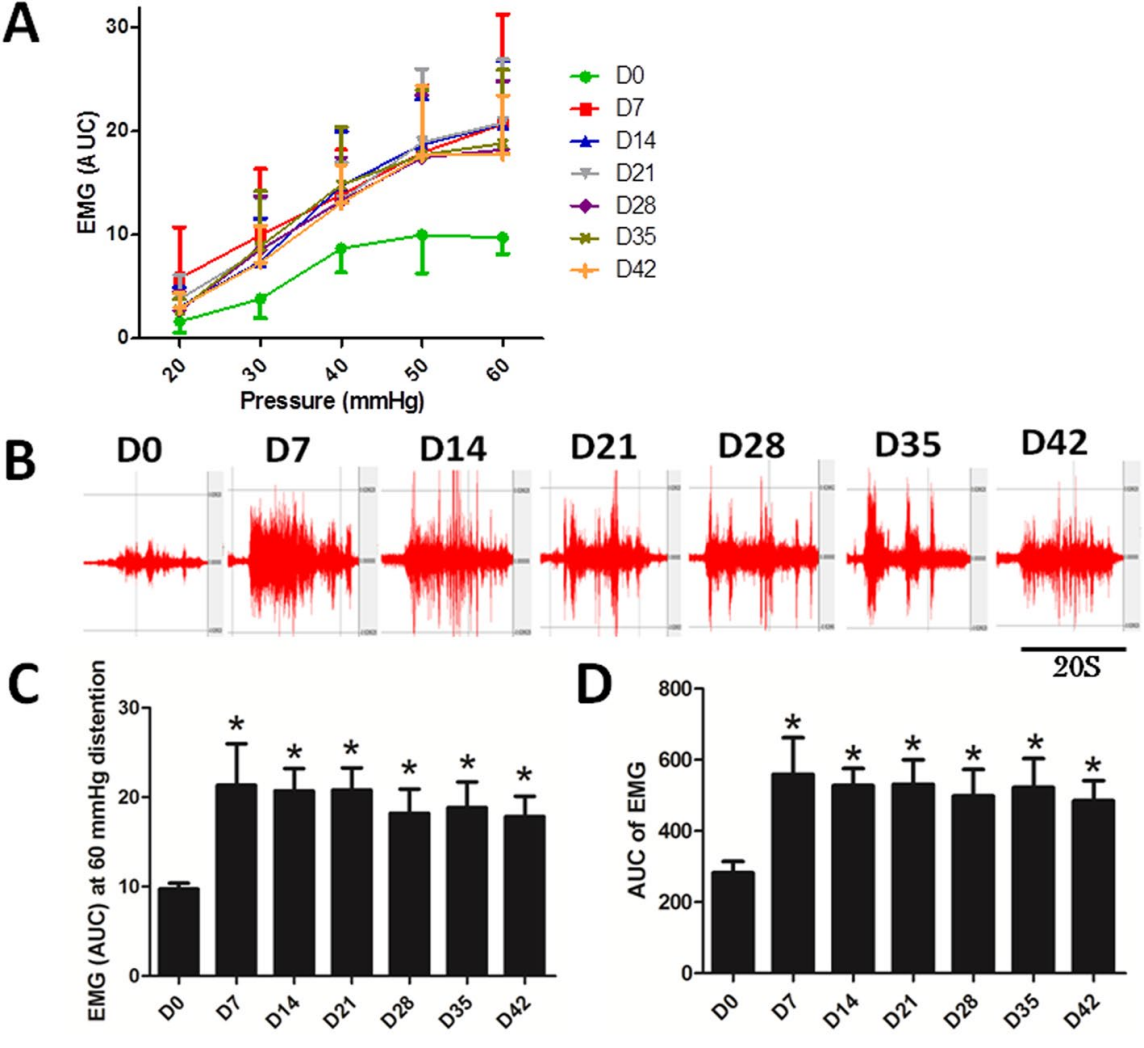
Abdominal EMG responses to colorectal distention in DSS-induced colitis rats. (**A**) The EMGs at different distention pressures and different time points. (**B**) Original EMG tracings at a colorectal distention pressure of 60 mmHg on different days in a typical rat. (**C**) The area under the curve (AUC) of the EMG at a distention pressure of 60 mmHg on different days. (**D**) The area under the curve (AUC) of the EMG summed at all distention pressures on different days. *P < 0.05 compared with Day 0. (N = 8).

Visceral hypersensitivity was noted during and after active inflammation. The visceromotor response to CRD assessed by the abdominal EMG was significantly increased from Day 7 to Day 42 at a rectal distention pressure of 40, 50 and 60 mmHg compared with Day 0 (Fig. 4A). As shown in the Fig. 4B,C, a significant difference was noted in the EMG from Day 7 to Day 42 during CRD at 60 mmHg when compared with Day 0 (*P* < 0.05, Day 7 to Day 42 vs. Day 0). The AUC of EMG curves summed over all distention pressures was also increased on Day 7, 14, 21, 28, 35 and 42, compared with Day 0 (all, *P* < 0.05 vs. Day 0) (Fig. 4D).

These findings demonstrated visceral hypersensitivity at all CRD pressures during active inflammation; most importantly, the DSS-induced visceral hypersensitivity persisted at least 5 weeks after the administration of DSS.

### Alterations in plasma ACh and NGF

**Figure 5:**
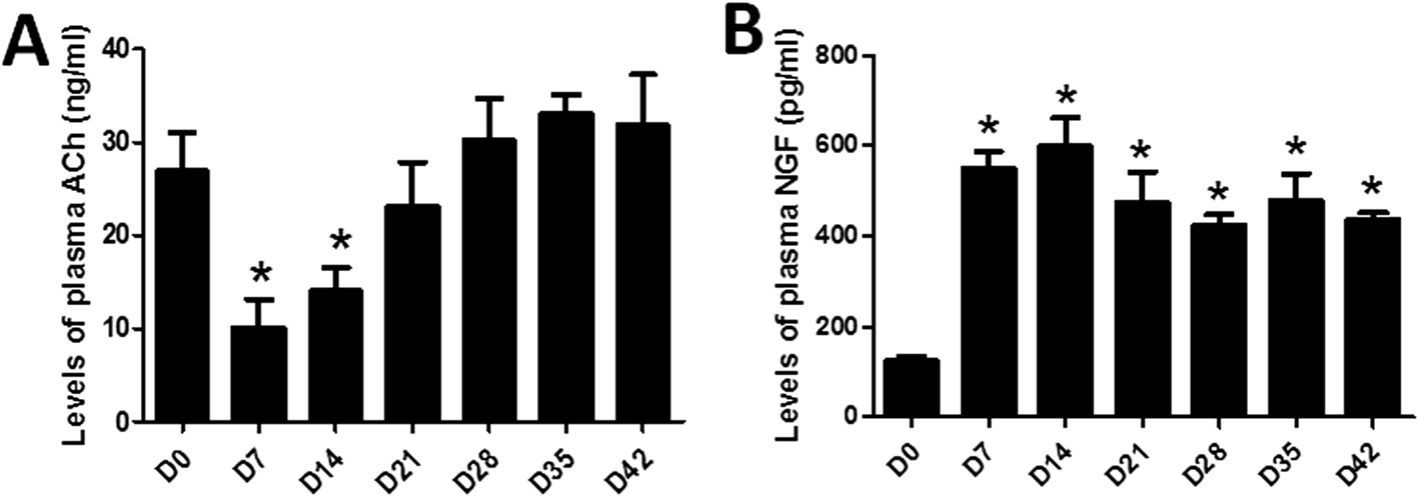
Levels of plasma acetylcholine (ACh) and nerve growth factor (NGF) in DSS-induced colitis rats. *P < 0.05 compared with Day 0. (N = 6).

As shown in Fig. 5A, the level of plasma ACh was decreased on Day 7 and Day 14 as compared to Day 0 (*P* = 0.003, *P* = 0.028). In the following days, the level of plasma ACh was gradually increased and became comparable to the baseline starting from Day 21 to Day 42 (all *P* > 0.05, vs. Day 0).

The plasma level of NGF is shown in Fig. 5B. It was increased dramatically during and after the DSS administration, compared to Day 0 (all *P* < 0.001).

### Correlation of NGF with EMG and ACh with WGTT

**Figure 6:**
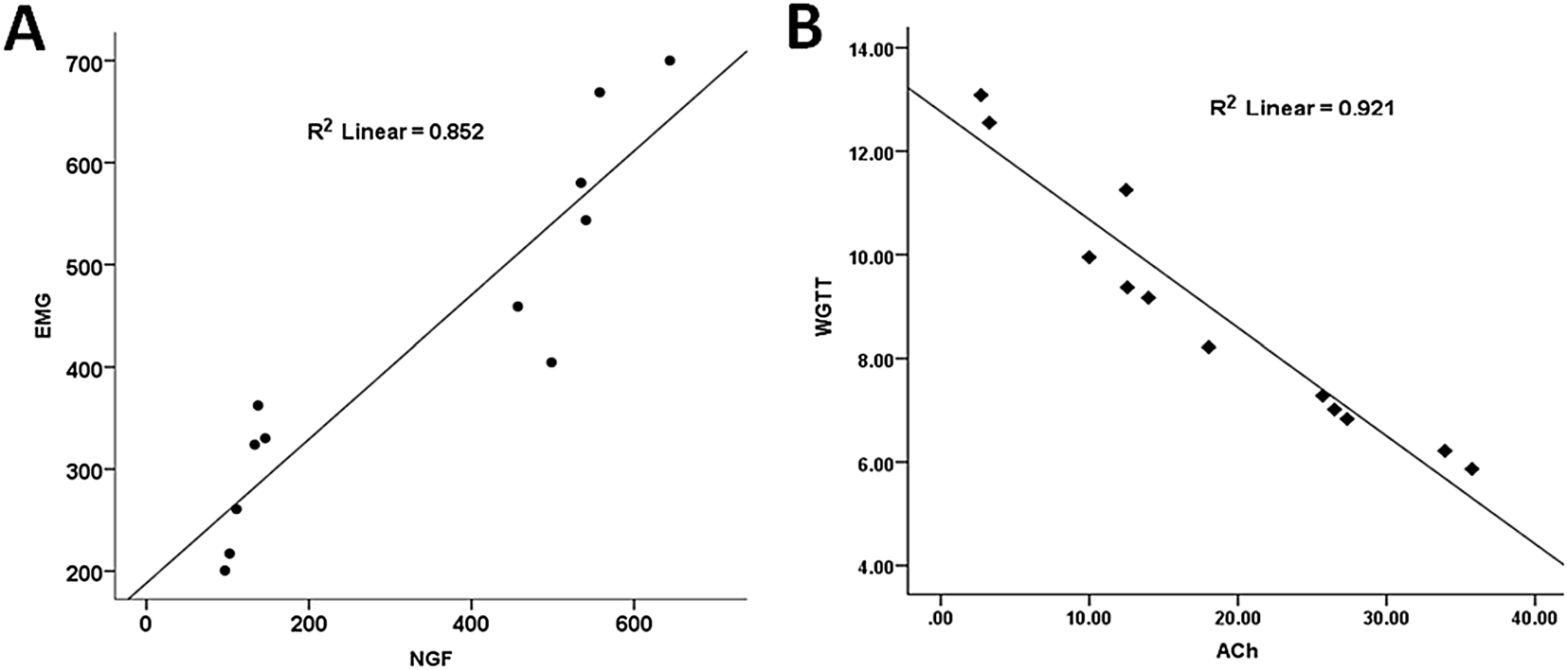
Correlations between plasma nerve growth factor (NGF) and the abdominal electromyogram (**A**) and between plasma acetylcholine (ACh) and the whole gut transit time (WGTT) (**B**) at baseline and one week after the DSS administration.

Pearson correlation and linear regression analyses revealed a significant positive correlation between plasma NGF and the abdominal EMG. The plasma level of NGF collected at baseline and on Day 7 was positively correlated with the AUC of the EMGs at all pressures assessed at baseline and on Day 7: r = 0.923 (*P* < 0.001, Pearson analysis). The linear regression analysis revealed following correlation between the plasma NGF level and the AUC of EMGs: R^2^ = 0.852, *P* < 0.001 (Fig. 6A). A significant negative correlation was noted between the plasma level of ACh and the WGTT obtained at baseline and on Day 7 after the DSS administration: r = 0.960, *P* < 0.001 (Pearson correlation analysis) and R^2^ = 0.921, *P* < 0.001 (linear regression analysis), indicating that the delayed whole gut transit on Day 7 was probably attributed to a decrease in plasma ACh (Fig. 6B).

## Discussion

In the present study, we have demonstrated that a 7-day administration of DSS induced colorectal inflammation that lasted for 2 more weeks after the termination of the administration. The DSS-induced inflammation resulted in visceral hypersensitivity that was present during and 6 weeks after the administration. Furthermore, visceral hypersensitivity was closely correlated with the plasma level of NGF that was dramatically increased during the DSS administration and remained high during the 6 weeks after the administration. Distal colon transit was accelerated during and after the DSS administration, whereas the whole gut transit was delayed during the DSS administration but accelerated after the administration. The delayed whole gut transit during the DSS administration was probably attributed to a decrease in plasma ACh.

UC is one of the two most common types of inflammatory bowel disease (IBD) that causes long-lasting inflammation, mainly in the colon. It has a chronic remitting and relapsing course with periods of asymptomatic remission interrupted with flares. In order to evaluate the inflammation, the DAI score, fecal calprotectin and plasma TNF-α were assessed in this study. The DAI score is frequently used to reflect the degree of inflammatory activity in animal and clinical studies^16,17^. The DAI score was reported to be significantly increased when drinking DSS water and gradually decreased after the administration of DSS^18^. A similar finding was noted in this study. Although the DAI score in the present study was assessed by two experienced researchers, there was still a need to objectively assess other measures of inflammation and we chose fecal calprotectin and TNF-α. Fecal calprotectin is not only used to assess IBD with a high sensitivity and specificity, but also applied to predict the clinical relapse and recurrence of IBD^19^. The increase in fecal calprotectin during and 2 weeks after the DSS treatment was observed in this study. TNF-α, a key pro-inflammatory mediator in UC patients, has been considered as a primary factor for pathologic inflammation. Plasma TNF-α in TNBS-induced colitis rats also showed that a dose of TNBS provoked a significant rise in the production of TNF-α and gradually decreased week by week^16^. In this study, all three biomarkers of inflammation, DAI score, fecal calprotectin and TNF-α, showed consistent results: the one-week DSS administration induced inflammation that lasted 2 more weeks after the administration and then it turned into low-level inflammation; starting from D28, no significant difference was noted in fecal calprotectin or TNF-α between the normal rats and rats treated with DSS. On Day 42, no difference was present in histology between the normal rats and rats treated with DSS.

As expected, visceral hypersensitivity was observed: the abdominal EMG responses to CRD at all tested distention pressures were elevated from Day 7 to Day 42. The novel finding was the significant correlation (r = 0.92 and *P* < 0.001) between the EMG and plasma NGF, suggesting an important role of NGF in the observed rectal hypersensitivity. As our analysis indicated, the EMG response to CRD could be predicted by the plasma level of NGF. NGF is required for the development and maintenance of the sympathetic and sensory nervous systems^20^. Barreau et al. reported NGF not only stimulated but also kept changes of visceral sensitivity induced by neonatal stress for a long time in rats^15^. It was also found that NGF was up-regulated in circular smooth muscles in rats with DSS-induced colitis^21^. Similarly, both the expressions of NGF and Trk high affinity receptor were increased in UC patients, indicating the activation of NGF/TrkR pathway in the chronic inflammation^22^. There is also a reported relationship between NGF and TNF-α, including that NGF promoted TNF-α expression in neurons and also TNF-α improved the expression of NGF in glial^23^; TNF-α was reported to initiate the synthesis and secretion of NGF in mouse and human fibroblasts^24^. Our results showed that the plasma NGF level was elevated dramatically from the beginning of the inflammation and persisted in high level. Through correlation and regression analyses, we found that the plasma NGF level was strongly and significantly correlated with visceral hypersensitivity, suggesting an important role of NGF.

Though colonic dysmotility leads to fecal urgency and fecal incontinence in patients with active and quiescent UC, rectal hypersensitivity also plays an important role in fecal urgency and fecal incontinence^25^. Rao et al. reported that patients with mild-to-moderate UC did not show a significant change in rectal sensitivity, possibly attributed to a small sample size and lack of patients with inactive and severe UC^9^. Loening-Baucke et al. showed that the rectal sensitivity and contractility were significantly increased in patients with active colitis and speculated that they could be related to active mucosal inflammation and ulceration^26^. Others found that symptoms such as pain and diarrhea in patients with UC in remission were associated with visceral hypersensitivity^12^; this was in the line with our findings of visceral hypersensitivity.

It is universally acknowledged that the colon motility function is influenced by inflammation^8^. Colon motility abnormalities were observed in patients with UC, such as rapid colon transit and increased propulsive activity^27^. The shortened distal colon transit time might be relevant to the symptoms of increased frequency of defecation, urgency and tenesmus. It was interesting to note that the whole gut transit was delayed during the daily administration of DSS but accelerated after the termination of the DSS administration. These findings suggested that the DSS-induced inflammation resulted in an acceleration of the whole gut transit that was sustained even after the resolution of inflammation. However, the daily administration of DSS delayed whole gut transit, attributed to the inhibitory effect of the DSS administration on ACh. This was supported by the strong and significant correlation between the whole gut transit time and the plasma level of ACh obtained before and after the 7-day administration of DSS. However, it should be noted that the alteration of plasma ACh is associated with a number of factors, such as the activities of the immune system^28^.

Impairment in gut motility during active inflammation was reported in a number of previous studies. A recent clinical study in UC patients demonstrated a prolonged gastrointestinal transit time during severe UC involving the severe inflammation of small intestine and colon^29^. Similarly, a study also suggested that the propulsive motility was impaired in the inflamed areas attributing to completely obstructed or temporarily halted in heavily inflamed regions^30^. Interestingly, some studies also illustrated increased colon motility during the remission of inflammation^31,32^. After the termination of the DSS administration, the prolonged whole gut transit with accelerated distal colon transit indicated that not only distal colon but also proximal colon/small intestine was affected by DSS-induced colitis, which was consistent with the previous study^33^. Further studies are needed to explore possible mechanisms involved in accelerated whole gut transit during the recovery phase of inflammation.

Rectal compliance is associated with the stiffness or distensibility of the rectal wall. Previous studies showed that rectal compliance was significantly decreased in patients with UC using anorectal manometry, suggesting a reduced distensibility of the rectum in UC^26^. Another study noted that rectal compliance was significantly decreased in patients with active and quiescent disease^34^. In this study, we used an electronic barostat (a gold-standard method) to assess the rectal compliance in rats treated with DSS. Our finding of a reduced compliance after the termination of the DSS administration was consistent with the previous studies. However, during the week of the DSS administration, rectal compliance was not altered, which was different from the previous studies. Studies in UC patients found that submucosal fibrosis was observed with histopathological changes of chronic mucosal injury, but not active inflammation^35,36^. Therefore, it was speculated that the reduced rectal compliance might attributed to the stiffness of the rectum caused by the inflammation and fibrosis of the rectal tissue that took time to develop and also to heal. The mucosal histopathological examination could explain why the rectal compliance was not altered during the first week but reduced from the second week and remained low until the end of the study (Day 42); unfortunately, it was not performed in the present study, which represents one limitation of the study. Actually, the stiffness of the colorectal wall can not only affect the compliance, but also result in motility abnormalities, leading symptoms such as fecal incontinence and diarrhea, even in the absence of inflammation.

In addition to the absence of the mucosal histopathological analysis at every point of time, there were a number of other limitations in this study. Firstly, there was a lack of mechanistic findings demonstrating the role of the enteric nervous system. Secondly, ACh was assessed from circulation rather than from colon tissue. ACh is produced by several cell types of the immune system and the blood levels shown here could also be related the activities of the immune system. Thirdly, the molecular mechanisms of altered motility and visceral sensitivity were not sufficiently studied; extra experiments should be done by inhibiting NGF or adding ACh to the rats to confirm the mechanisms. Finally, this study was terminated at 6 weeks after the DSS administration, the follow up of acute DSS colitis could be extended to observe some alterations in a chronic phase.

In conclusion, DSS-induced inflammation leads to visceral hypersensitivity that is sustained until the resolution of inflammation and is probably mediated by NGF. Rectal compliance is reduced one week after the DSS-induced inflammation and the reduction is sustained until the resolution of inflammation. Distal colon transit is accelerated during and after resolution of active inflammation. Whole gut transit is accelerated one week after DSS-induced inflammation; it is, however, delayed during daily DSS administration, probably attributed to the DSS-induced decrease in plasma acetylcholine.

## Materials and methods

### Animals

Male Sprague Dawley rats (N = 16) weighted 200–250 g were obtained from Charles River Laboratories (MD, USA) and housed under standard normal laboratory conditions (22–23℃, 12/12 h light–dark cycle) with food and water ad libitum. After one week to accommodate to the environment, the rats were used for the study and randomly divided into the normal rats (n = 6) and the DSS-administrated rats (n = 10). Protocols for ethical use of animals were approved by the Animal Care and Use Committee of the Johns Hopkins University. All experiments were performed in accordance with the ACUC guidelines of the Johns Hopkins University. The study was carried out in compliance with the ARRIVE guidelines.

### Electromyogram (EMG) electrode placement

The rat was anesthetized with 1.5% isoflurane inhalation (Abbott Laboratories, North Chicago, IL, USA). Vital signs, including temperature and respiration rate, were monitored throughout the entire surgical process. A heating pad was used to maintain a constant body temperature at about 37 ± 0.5 °C. The anesthesia depth was assessed by hind paw pinch and respiration rate.

After the rat was anesthetized, an abdominal incision of about 2 cm was made. One pair of cardiac pacing wires (A&E Medical, Farmingdale, NJ) used as electrodes for EMG recording, were fixed in the external oblique muscles of the abdomen^37^. The electrodes were spaced 0.5 cm apart, and the connecting wires were tunneled underneath the skin and externalized at the back of the neck. After the surgery, the rat was placed in an individual cage in order to prevent the externalized electrode sires being chewed off by the other rat. Rats were given 5 days for a complete recovery before the experiment was initiated.

### Experimental protocol

**Figure 7:**
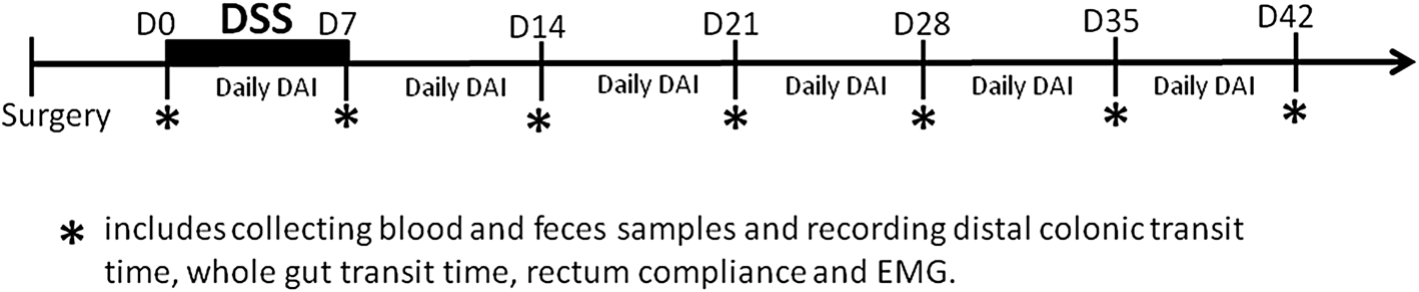
Flow chart of the experiment. After surgery for electrode implantation, 5% DSS water was given to the rats. The distal colon transit time (dCTT), whole gut transit time (WGTT), rectal compliance and EMG were measured with feces and blood samples collected every 7 days. Body weight and feces were recorded daily for the DAI score.

Dextran sulphate sodium (DSS) was used to induce ulcerative colitis^38^. The rats were given drinking water with 5% DSS (molecular weight 40 kD; Alfa Aesar, CA) for 7 days^39^. After that, the DSS rats were provided with regular drinking water without DSS. The normal rats were only given regular drinking water. During and after the treatment of DSS, the DAI score was recorded daily, dCTT, WGTT, rectal compliance, abdominal EMG, feces and blood samples were assessed/collected weekly for a period of 7 weeks (Days 0, 7, 14, 21, 28, 35, and 42) (Fig. 7).

### Evaluation of DAI

Animals were observed by two experienced researchers at least once daily for the activity, water/food consumption, stool consistency, and the presence of gross blood in feces and epic-anus. According to a previous study, the DAI score was assessed by weight loss, stool consistency, and bleeding^40^. The score was calculated as the total of the three components: (1) weight loss: no loss as 0 score; 5–10% as 1 score; 10–15% as 2 score; 15–20% as 3 score; and 20% as 4 score; (2) stool: normal as 0 score; loose stool as 2 score; diarrhea as 4 score; (3) bleeding: no blood as 0 score; presence as 2 score; gross blood as 4 score. The DAI score was recorded daily during the study.

### ELISA for fecal calprotectin, TNF-α, ACh and NGF

Fecal samples were stored at − 80 °C until analysis. An ELISA kit (MyBiosource, San Diego, USA) was used to measure the levels of fecal calprotectin following the manufacturer’s instructions. The absorbance was measured at 450 nm.

Blood samples were collected from the tail vein and centrifuged at 3000 rpm for 15 min, and then the plasma was stored at − 80 °C before analysis. Plasma TNF-α, ACh and NGF were quantified by corresponding ELISA kits (Abcam, Cambridge, UK; MyBiosource, San Diego, USA; Boster Biological Technology, Pleasanton, USA) according to the manufacturers’ protocols. The optical density (OD) was measured on an ELISA plate scanner.

### Histological analysis

The distal colon tissue was obtained and fixed in paraformaldehyde for 24 h. After dehydrated in graded ethanol and embedded in paraffin wax blocks, slices were made to do hematoxylin and eosin staining. The histological scores were achieved according to architectural derangements, inflammatory cell infiltration, goblet cell depletion and ulceration^40^.

### Distal colonic transit time (dCTT)

The dCTT was assessed as described previously with slight modificationsx^41^. After an overnight fast, the rat was inserted with a 5-mm glass bead 3 cm from the anus using a plastic rod and then placed in an empty transparent cage to observe the expulsion of the bead. The dCTT was defined as the time interval from the bead insertion to the bead expulsion.

### Whole gut transit time (WGTT)

The WGTT was defined as the time interval between the phenol red injection into the stomach and the first appearance of phenol red in the feces^41^. At 9 o’clock, the rat was gavaged with 3 ml 0.5% phenol red and then kept in an individual cage with normal feeding and drinking. About 13 o’clock, the color of the feces was observed carefully at an interval of 10 min. 0.1 N NaOH was used to improve the maximum color development of phenol red. The time of the first appearance of phenol red in the feces was used to determine the WGTT.

### Measurement of rectal compliance

Rectal compliance represents the pressure–volume relationship and reflects the elasticity of the rectum. Reduced rectal compliance is believed to contribute to diarrhea. It was assessed by the isobaric phasic distention of the rectum via an intra-rectal balloon that was connected and controlled by a computerized barostat device (Distender Series, Toronto, Canada). In order to reduce variability in compliance, a conditioning distention was started from 0 to 20 mmHg with a stepwise increment of 4 mmHg. Each distention level was maintained for 20 s. For the measurement of rectal compliance, it was performed from 0 to 20 mmHg with a step size of 2 mmHg. Each level of distention was also maintained for 20 s with 1 min intervals^42,43^.

### Measurement and analysis of EMG

The EMG activity reflects the contraction of the external oblique muscle in response to colorectal distention (CRD). A balloon was inserted into the colorectum 5 cm from the anal verge. The balloon was then distended sequentially at a pressure of 20, 30, 40, 50 and 60 mmHg and maintained for 20 s with a 4 min interval between two distention pressures. The EMG was recorded with a frequency range of 10 to 5000 Hz using an EMG amplifier (EMG 100C; Biopac systems, Inc, Santa Barbara, CA, USA). The area under the curve (AUC) of EMG was calculated by the software (Acknowledge; Biopac System, Inc., Santa Barbara CA). The final EMG data were presented as a percent increase against the baseline value before each distention: the AUC of the EMG activity during the 20 s distention divided by the 20 s baseline was defined as the EMG response to CRD^44^.

### Statistical analysis

Statistical analyses were performed using SPSS version 24.0 for Windows (SPSS, Chicago, IL, USA). The results are presented as the means ± SEM. Analysis of Variance was used for assessing the difference among different time points. Comparisons were then carried out by LSD test as the variance was equal and by Dunnett’s T3 test when equal variance was not assumed. Values of *P* less than 0.05 were considered as statistically significant. Pearson correlations and linear regressions were performed to determine the relationship between NGF and EMG and between ACh and WGTT.

## Data Availability

The data used to support the findings of this study are available on request from the authors.
